# Supplementary material for: A Novel Strategy for Screening Tumor-Specific Variable Domain of Heavy-Chain Antibodies
Source: Int J Mol Sci. 2023 Jun 28;24(13):10804. doi: 10.3390/ijms241310804 (PMC10341490; doi:10.3390/ijms241310804)
Supplement: Supplementary file 1 [file ijms-24-10804-s001.zip › ijms-2424776-supplementary.pdf]

Supplementary Materials

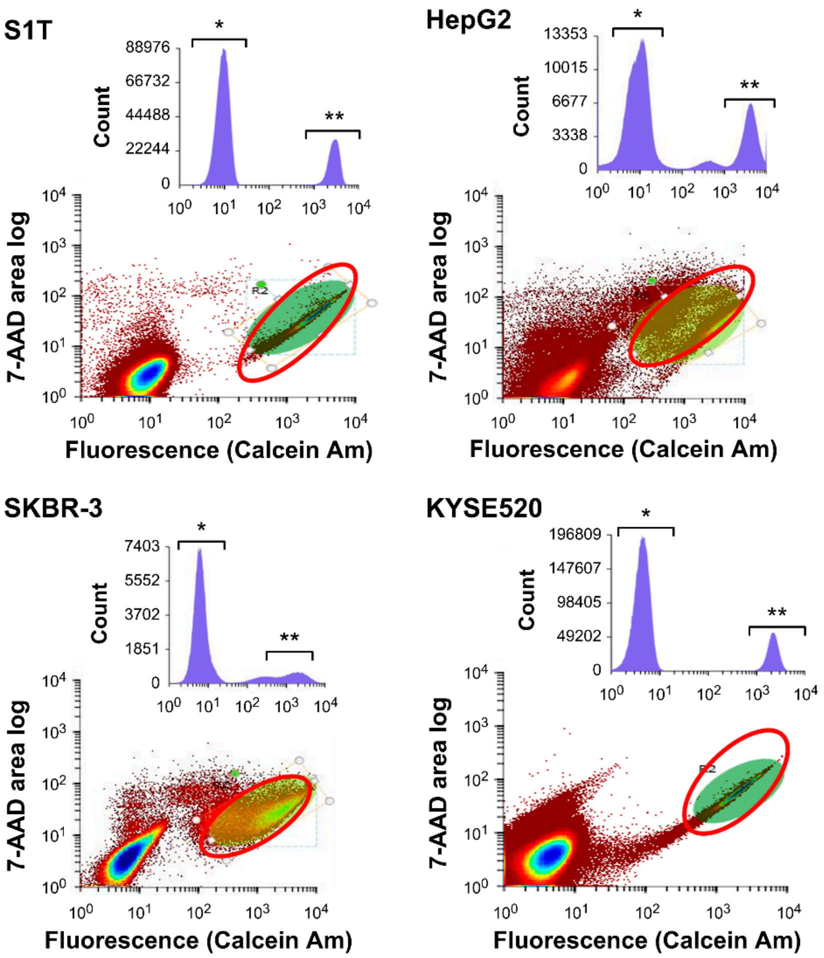

Figure S1. Phage library enrichment using fluorescence-activated cell sorting (FACS). The calcein-AM stained target cancer cells were separated from the absorbent cells, indicated by red circles. The remaining high-density populations near the baseline were absorbent cells. The Y-axis in the lower graph indicates the 7-AAD area log to focus on dead cells, while the X-axis indicates the fluorescence (calcein-AM) area log. The red circles represent the cells that rescue the binding phages. The upper panel focuses on the histogram of absorbent cells and calcein-AM-reacted target cells for each diagram. The single asterisk (*) indicates the absorbent cells, while the double asterisk (**) indicates calcein-AM-stained target cells. The absorbent cells were three cancer cell lines.

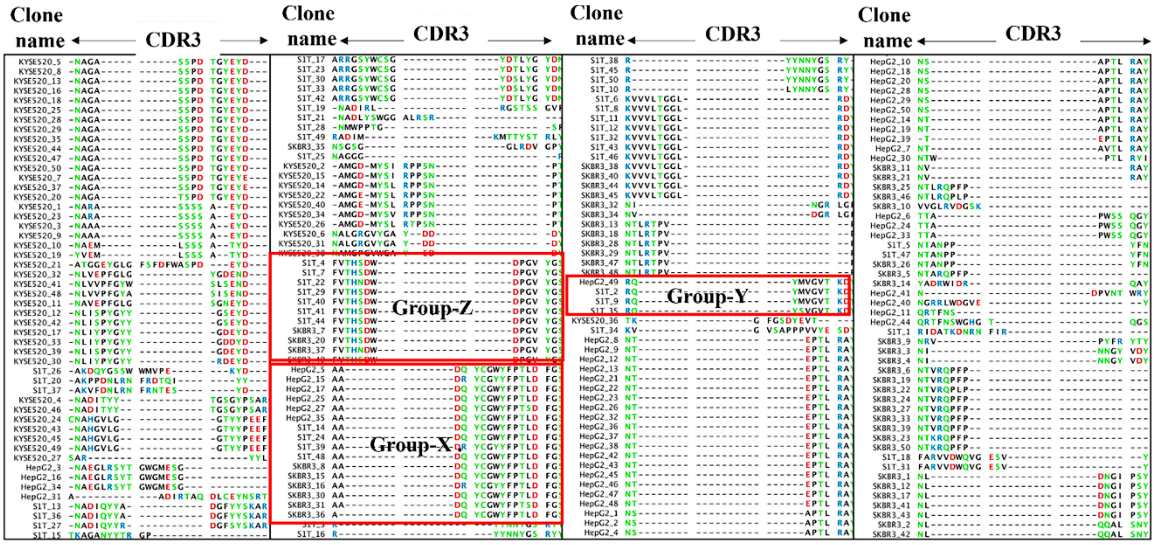

Figure S2. Alignment of the combined 200 VHH clone CDR3 sequences. A total of 200 VHH clones (top50 VHH clones from each cell-line library) with high amplification factors were aligned using ClustalW. The library names and clone numbers were assigned at the beginning of each CDR3 amino acid sequence. The asterisk (*) indicates unique CDR3, derived from different libraries. The different colors of the characters in the sequences reflect the properties of the amino acids. These NGS data sequences were deposited in the DDBJ Sequence Read Archive (DRA) under accession number DRA014254.

Table S1. Frequency (%) and amplification factor of NGS identified top 50 sequences for each library.

Clones Name	0R frequency (%)	2R frequency (%)	Amplification factor	Clone name	0R frequency (%)	2R frequency (%)	Amplification factor	Clone name	0R frequency (%)	2R frequency (%)	Amplification factor	Clone name	0R frequency (%)	3R frequency (%)	Amplification factor
S1T-1	0.00077	3.5434	4601.79	SKBR3-1	0.00086	11.5050	13447.25	HepG2-1	0.00086	1.5575	1820.39	KYSE520-1	0.00086	6.2176	7267.25
S1T-2	0.00077	3.2755	4253.91	SKBR3-2	0.00077	7.7596	10077.37	HepG2-2	0.00171	2.9427	1719.76	KYSE520-2	0.00086	5.1733	6046.69
S1T-3	0.00077	3.1517	4093.19	SKBR3-3	0.00077	1.9021	2470.24	HepG2-3	0.00086	0.7511	877.87	KYSE520-3	0.00077	2.3040	2992.18
S1T-4	0.00077	2.5233	3277.06	SKBR3-4	0.00077	1.4019	1820.66	HepG2-4	0.00171	1.2855	751.29	KYSE520-4	0.00077	1.4389	1868.73
S1T-5	0.00171	2.4644	1440.23	SKBR3-5	0.00077	0.7739	1005.13	HepG2-5	0.00077	0.4862	631.38	KYSE520-5	0.00086	0.8373	978.69
S1T-6	0.00085	1.2166	1422.07	SKBR3-6	0.00086	0.8366	977.90	HepG2-6	0.00077	0.4667	606.09	KYSE520-6	0.00086	0.7861	918.85
S1T-7	0.00085	1.1550	1350.06	SKBR3-7	0.00077	0.7170	931.15	HepG2-7	0.00077	0.4612	599.00	KYSE520-7	0.00171	1.4176	828.46
S1T-8	0.00077	0.8877	1152.88	SKBR3-8	0.00077	0.7141	927.43	HepG2-8	0.00077	0.4379	568.65	KYSE520-8	0.00171	1.3888	811.63
S1T-9	0.00077	0.6284	816.13	SKBR3-9	0.00077	0.5983	777.00	HepG2-9	0.00342	1.9408	567.11	KYSE520-9	0.00077	0.5931	770.21
S1T-10	0.00077	0.4725	613.66	SKBR3-10	0.00077	0.5203	675.68	HepG2-10	0.00077	0.4184	543.35	KYSE520-10	0.00077	0.5664	735.58
S1T-11	0.00077	0.4698	610.19	SKBR3-11	0.00077	0.5126	665.73	HepG2-11	0.00513	2.1854	425.73	KYSE520-11	0.00086	0.6016	703.16
S1T-12	0.00171	1.0104	590.50	SKBR3-12	0.00077	0.4446	577.46	HepG2-12	0.00077	0.3140	407.77	KYSE520-12	0.00257	1.7440	679.47
S1T-13	0.00085	0.4285	500.95	SKBR3-13	0.00077	0.4370	567.52	HepG2-13	0.12833	47.820	372.62	KYSE520-13	0.00086	0.5632	658.28
S1T-14	0.00077	0.3434	445.98	SKBR3-14	0.00077	0.4288	556.95	HepG2-14	0.00257	0.8547	332.99	KYSE520-14	0.00077	0.4629	601.21
S1T-15	0.00077	0.3187	413.98	SKBR3-15	0.00077	0.3949	512.82	HepG2-15	0.00077	0.2220	288.37	KYSE520-15	0.00077	0.4256	552.72
S1T-16	0.00077	0.3096	402.15	SKBR3-16	0.00077	0.3609	468.68	HepG2-16	0.00941	2.4573	261.11	KYSE520-16	0.00086	0.4053	473.76
S1T-17	0.00077	0.2909	377.80	SKBR3-17	0.00077	0.3480	451.90	HepG2-17	0.00077	0.1807	234.74	KYSE520-17	0.00077	0.3605	468.22
S1T-18	0.00077	0.2813	365.28	SKBR3-18	0.00077	0.3207	416.47	HepG2-18	0.00086	0.1963	229.48	KYSE520-18	0.00077	0.3168	411.42
S1T-19	0.00085	0.3091	361.31	SKBR3-19	0.04877	20.128	412.75	HepG2-19	0.00077	0.1449	188.20	KYSE520-19	0.00077	0.2816	365.71
S1T-20	0.00077	0.2754	357.62	SKBR3-20	0.00086	0.3499	408.95	HepG2-20	0.00342	0.5781	168.92	KYSE520-20	0.00086	0.3093	361.55
S1T-21	0.00077	0.2513	326.31	SKBR3-21	0.00077	0.3020	392.23	HepG2-21	0.00086	0.1433	167.56	KYSE520-21	0.00077	0.2773	360.17
S1T-22	0.00171	0.5357	313.09	SKBR3-22	0.00171	0.5011	292.87	HepG2-22	0.00077	0.1161	150.76	KYSE520-22	0.00077	0.2453	318.61
S1T-23	0.00077	0.2352	305.44	SKBR3-23	0.00171	0.4911	286.99	HepG2-23	0.00171	0.2470	144.34	KYSE520-23	0.00077	0.2432	315.84
S1T-24	0.00077	0.2288	297.09	SKBR3-24	0.00428	1.2109	283.08	HepG2-24	0.00086	0.1153	134.78	KYSE520-24	0.02396	7.4027	309.01
S1T-25	0.00257	0.7468	290.97	SKBR3-25	0.00513	1.4148	275.62	HepG2-25	0.00086	0.1067	124.76	KYSE520-25	0.00077	0.2368	307.53
S1T-26	0.00077	0.2170	281.78	SKBR3-26	0.00171	0.4403	257.34	HepG2-26	0.00077	0.0834	108.27	KYSE520-26	0.00077	0.2336	303.37
S1T-27	0.00427	1.1117	259.87	SKBR3-27	0.00684	1.6259	237.55	HepG2-27	0.00342	0.3498	102.22	KYSE520-27	0.00077	0.2315	300.60
S1T-28	0.00077	0.1907	247.69	SKBR3-28	0.00086	0.1876	219.30	HepG2-28	0.00077	0.0771	100.17	KYSE520-28	0.00513	1.4944	291.11
S1T-29	0.00085	0.2009	234.82	SKBR3-29	0.02823	5.9126	209.42	HepG2-29	0.00077	0.0717	93.09	KYSE520-29	0.00086	0.2389	279.27
S1T-30	0.00077	0.1795	233.08	SKBR3-30	0.00086	0.1728	201.96	HepG2-30	0.00171	0.1543	90.15	KYSE520-30	0.00171	0.4757	278.02
S1T-31	0.00077	0.1746	226.82	SKBR3-31	0.00342	0.6748	197.20	HepG2-31	0.00086	0.0763	89.24	KYSE520-31	0.00171	0.4299	251.22
S1T-32	0.00077	0.1687	219.17	SKBR3-32	0.00086	0.1680	196.36	HepG2-32	0.00077	0.0615	79.93	KYSE520-32	0.00077	0.1845	239.65
S1T-33	0.00085	0.1779	207.89	SKBR3-33	0.00770	1.4818	192.45	HepG2-33	0.00077	0.0600	77.91	KYSE520-33	0.00086	0.2048	239.37
S1T-34	0.00077	0.1452	188.55	SKBR3-34	0.00077	0.1479	192.07	HepG2-34	0.00077	0.0600	77.91	KYSE520-34	0.00077	0.1749	227.18
S1T-35	0.00077	0.1382	179.51	SKBR3-35	0.00086	0.1618	189.09	HepG2-35	0.00077	0.0584	75.89	KYSE520-35	0.02909	6.5952	226.72
S1T-36	0.06930	12.039	173.72	SKBR3-36	0.00077	0.1292	167.83	HepG2-36	0.00077	0.0584	75.89	KYSE520-36	0.00342	0.7627	222.85
S1T-37	0.00077	0.1318	171.16	SKBR3-37	0.00171	0.2623	153.28	HepG2-37	0.00077	0.0576	74.87	KYSE520-37	0.00077	0.1707	221.64
S1T-38	0.00077	0.1318	171.15	SKBR3-38	0.00086	0.1292	151.05	HepG2-38	0.00086	0.0639	74.67	KYSE520-38	0.00077	0.1632	211.95
S1T-39	0.00077	0.1275	165.59	SKBR3-39	0.00171	0.2388	139.58	HepG2-39	0.00077	0.0561	72.85	KYSE520-39	0.00077	0.1589	206.40
S1T-40	0.00077	0.1264	164.20	SKBR3-40	0.00077	0.1048	136.13	HepG2-40	0.00077	0.0553	71.84	KYSE520-40	0.00171	0.3296	192.62

S1T-41	0.00077	0.1216	157.94	SKBR3-41	0.00077	0.1024	133.02	HepG2-41	0.00086	0.0608	71.03	KYSE520-41	0.00077	0.1419	184.24
S1T-42	0.04021	5.9425	147.78	SKBR3-42	0.00077	0.0981	127.43	HepG2-42	0.00086	0.0608	71.03	KYSE520-42	0.00428	0.7595	177.53
S1T-43	0.00077	0.1125	146.11	SKBR3-43	0.00077	0.0952	123.70	HepG2-43	0.00077	0.0537	69.82	KYSE520-43	0.00077	0.1365	177.31
S1T-44	0.00077	0.1093	141.93	SKBR3-44	0.00077	0.0756	98.21	HepG2-44	0.00077	0.0530	68.80	KYSE520-44	0.00077	0.1344	174.54
S1T-45	0.00077	0.1082	140.54	SKBR3-45	0.00171	0.1503	87.83	HepG2-45	0.00077	0.0514	66.78	KYSE520-45	0.00077	0.1259	163.46
S1T-46	0.00077	0.1012	131.50	SKBR3-46	0.00086	0.0742	86.71	HepG2-46	0.00077	0.0506	65.77	KYSE520-46	0.00077	0.1259	163.46
S1T-47	0.00077	0.1007	130.80	SKBR3-47	0.00086	0.0737	86.15	HepG2-47	0.00077	0.0491	63.74	KYSE520-47	0.00077	0.1237	160.69
S1T-48	0.00077	0.0996	129.41	SKBR3-48	0.00077	0.0656	85.16	HepG2-48	0.00077	0.0491	63.74	KYSE520-48	0.00086	0.1355	158.33
S1T-49	0.00085	0.1018	118.97	SKBR3-49	0.00086	0.0713	83.35	HepG2-49	0.00077	0.0483	62.73	KYSE520-49	0.00077	0.1205	156.53
S1T-50	0.00257	0.3048	118.77	SKBR3-50	0.00171	0.1407	82.24	HepG2-50	0.00077	0.0483	62.73	KYSE520-50	0.00077	0.1184	153.76

Table S2. List of designed CDR3-specific primer sets for overlapping PCR.

Group Name	Forward Primer	Reverse Primer	nt	Tm (°C)	GC (%)
Group A	5′-TGTAATTCGGCGCCGACGCT-3′	5′-AGCGTCGGCGCCGAATTACA-3′	20	62.3	60
Group B	5′-GGATCGCGTTACTACGGCC-3′	5′-GGCCGTAGTAACGCGATCC-3′	19	58	63.2
Group C	5′-TGCAATACCCTTCGGACGCCAGTA-3′	5′-TACTGGCGTCCGAAGGGTATTGCA-3′	24	62.5	54.2
Group D	5′-TGTAATACCGTAAGGCAACCCTTT-3′	5′-AAAGGGTTGCCTTACGGTATTACA-3′	24	56.7	41.7
Group E	5′-TGTGCAATGGGGGACATGTACTCT-3′	5′-AGAGTACATGTCCCCCATTGCACA-3′	24	60.3	50
Group F	5′-TGTAATCTACAGCAAGCGCTTTCG-3′	5′-CGAAAGCGCTTGCTGTAGATTACA-3′	24	58.5	45.8
Group G	TGTAATGCCCGAGCGAGTAGTAGT-3′	5′-ACTACTACTCGCTCGGGCATTACA-3′	24	59.7	50
Group X	5′-TGCAGCAGATCAATATTGCGGCTG-3′	5′-CAGCCGCAATATTGATCTGCTGCA-3′	24	60.8	50
Group Y	5′-TCGTCAATACATGGTTGGGGTCAC-3′	5′-GTGACCCCAACCATGTATTGACGA-3′	24	59.4	50
Group Z	5′-TGTTTCGTCACTCATAGCGACTGG-3′	5′-CCAGTCGCTATGAGTGACGAAACA-3′	24	59.4	50
